# PTHrP Induces Autocrine/Paracrine Proliferation of Bone Tumor Cells through Inhibition of Apoptosis

**DOI:** 10.1371/journal.pone.0019975

**Published:** 2011-05-23

**Authors:** Isabella W. Y. Mak, Robert W. Cowan, Robert E. Turcotte, Gurmit Singh, Michelle Ghert

**Affiliations:** 1 Department of Surgery, McMaster University, Hamilton, Ontario, Canada; 2 Department of Medical Sciences, McMaster University, Hamilton, Ontario, Canada; 3 Department of Orthopaedic Surgery, McGill University Health Centre, Montreal General Hospital, Montreal, Quebec, Canada; 4 Department of Pathology and Molecular Medicine, McMaster University, Hamilton, Ontario, Canada; 5 Juravinski Cancer Centre, Hamilton Health Sciences, Hamilton, Ontario, Canada; Centro Cardiologico Monzino, Italy

## Abstract

Giant Cell Tumor of Bone (GCT) is an aggressive skeletal tumor characterized by local bone destruction, high recurrence rates and metastatic potential. Previous work in our lab has shown that the neoplastic cell of GCT is a proliferating pre-osteoblastic stromal cell in which the transcription factor Runx2 plays a role in regulating protein expression. One of the proteins expressed by these cells is parathryroid hormone-related protein (PTHrP). The objectives of this study were to determine the role played by PTHrP in GCT of bone with a focus on cell proliferation and apoptosis. Primary stromal cell cultures from 5 patients with GCT of bone and one lung metastsis were used for cell-based experiments. Control cell lines included a renal cell carcinoma (RCC) cell line and a human fetal osteoblast cell line. Cells were exposed to optimized concentrations of a PTHrP neutralizing antibody and were analyzed with the use of cell proliferation and apoptosis assays including mitochondrial dehydrogenase assays, crystal violet assays, APO-1 ELISAs, caspase activity assays, flow cytometry and immunofluorescent immunohistochemistry. Neutralization of PTHrP in the cell environment inhibited cell proliferation in a consistent manner and induced apoptosis in the GCT stromal cells, with the exception of those obtained from a lung metastasis. Cell cycle progression was not significantly affected by PTHrP neutralization. These findings indicate that PTHrP plays an autocrine/paracrine neoplastic role in GCT by allowing the proliferating stromal cells to evade apoptosis, possibly through non-traditional caspase-independent pathways. Thus PTHrP neutralizing immunotherapy is an intriguing potential therapeutic strategy for this tumor.

## Introduction

Giant Cell Tumor of Bone (GCT) is an aggressive and highly osteolytic bone tumor that is characterized by local osteolysis, regional pain and the predisposition to pathological fracture [1]. Current preferred treatment of GCT consists of limb sparing surgery by the means of extended curettage with the addition of local adjuvant therapies [2], [3]. Albeit anatomy and function are preserved with such an approach, local recurrence rates remain high [4], thus emphasizing the importance of developing an understanding of the biology of this tumor and subsequent creation of more effective therapeutic options.

The cellular elements of GCT include both osteoclast-like giant cells and proliferating osteoblast-like stromal cells [5]. Previous work in our lab has shown that the osteoblastic transcription factor Runx2 and AP-1 plays an important role in regulating protein expression in the neoplastic cells stromal cells of GCT. [6], [7], [8], [9]. Among these proteins, we have found that parathyroid hormone-related protein (PTHrP) and its receptor are constitutively expressed in this tumor [10]. In some pathways, such as the Indian hedgehog (Ihh) pathway, Runx2 and PTHrP have been shown to regulate each other in a reciprocal fashion [11], [12], [13].

PTHrP is present in many organs and tissues exerting its effects through an autocrine/paracrine action [14]. PTHrP shares the same N-terminal end as parathyroid hormone (PTH); therefore, it can simulate most of the actions of PTH including increases in bone resorption [15]. PTHrP was first identified as the tumor-derived agent responsible for humoral hypercalcemia of malignancy [16]. When produced in prodigious amounts by tumors, PTHrP, by virtue of its ability to bind to and activate the G protein–coupled PTH/PTHrP receptor, is the humoral factor responsible for marked bone resorption and hypercalcemia [17], [18].

The majority of neoplastic tissues that metastasizes to bone produce PTHrP, and PTHrP expression correlates with skeletal localization of tumors [19]. Therefore, PTHrP is an intriguing therapeutic target in the setting of malignancy, particularly in the bone microenvironment. In fact, a recent study demonstrated the anti-proliferative effect of PTHrP neutralizing antibody in human renal cell carcinoma *in vitro* and *in vivo* [20]. The objectives of this study were to determine if PTHrP plays a role in cellular proliferation in GCT, and if so, to identify the underlying mechanism of neoplastic proliferation provided by PTHrP.

## Materials and Methods

### Ethics statement

The use of all patient-derived material was approved by the Hamilton Health Sciences and McMaster University Faculty of Health Sciences Research Ethics Board (REB Project #: 05-302). Written patient informed consent was obtained individually. The Hamilton Health Sciences/McMaster University Research Ethics Board operates in compliance with the ICH Good Clinical Practice Guidelines and the Tri-Council Policy Statement: Ethical Conduct for Research Involving Human and Division 5 Health Canada Food and Drug Regulations.

### GCT sample collection

The diagnosis of GCT of bone was established by biopsy prior to surgical excision. Specimens were obtained at the time of surgery from patients undergoing tumor resection and a bone pathologist verified the diagnosis of GCT post-operatively. Tissue samples from five cases of GCT of bone were used in this study and all experiments were performed in triplicate or as otherwise stated for all five patient specimens.

### Primary cell lines and cultures

Primary cell cultures of GCT stromal tumor cells were isolated, characterized and established from fresh GCT tissues as described in our previous studies [7]. The specimens were freshly minced in Dulbecco's Modified Eagle Medium (D-MEM, Gibco, Burlington, ON) producing a cell suspension with small fragments of tissue. The resultant suspension was passed through a 20-gauge needle prior to seeding in cell culture flasks with D-MEM supplemented with 10% fetal bovine serum (FBS), 2 mM L-glutamine, 100 U/mL penicillin, and 100 µg/mL streptomycin (Gibco). The cell suspension, together with macerated tissue, was cultured in 37°C humidified air with 5% CO2. Culture medium was changed every two to three days until ∼80% confluence. Confluent cells (∼80%) were subcultured after dissociating with trypsin and ethylenediaminetetraacetic acid (EDTA). Following several successive passages, the mesenchymal stromal cells became the predominant cell type whereas the multinucleated giant cells were eliminated from the culture. Primary cultures of the proliferating homogenous stromal tumor cell population obtained after the fifth or sixth passage (without any hematopoietic markers) and up to the tenth passage were used for experiments.

Human fetal osteoblast (hFOB) 1.19 cells (American Type Culture Collection, ATCC#CRL-11372) and renal cell adenocarcinoma CRL1932 (Designation: 786-O, American Type Culture Collection, ATCC#CRL-1932) were used as control cell lines. hFOB cells were maintained in supplemented DMEM/F-12 medium at 33.5oC. CRL1932 cells were maintained in supplemented RPMI-1640. CRL1932 cells, as a positive control, are known to produce PTHrP that is identical to peptides produced by breast and lung tumors [21].

### RNA purification and reverse transcription (RT)

Total RNA was isolated from GCT stromal cells using the RNeasy mini kit (Qiagen, ON) as optimized in our lab. To ensure complete removal of contaminating genomic DNA prior to first-strand synthesis, RNase-free DNase I treatment was applied on the RNeasy column during total RNA isolation. Single-stranded complementary DNA (cDNA) was synthesized from 1.0 µg of total RNA using the SuperScripts III First-Strand Synthesis System for RT-PCR (Invitrogen) and oligo(dT) 12–18 primer, following the manufacturer's instructions.

### PCR and real-time polymerase chain reaction (PCR)

The expression of GAPDH and PTHrP was analyzed using real-time RT-PCR. In brief, real-time PCR analysis was performed on cDNA synthesized from GCT stromal cell total RNA using the MiniOpticon Real-Time PCR Detection System with the iQ SYBR Green Supermix (Bio-Rad Laboratories, ON) according to the manufacturer's instructions. Cycling consisted of 40 cycles of 15 s at 95°C, 30 s at 58°C, and 30 s at 72°C, operated with the Opticon Monitor software v3.1. PCR experiments were performed in triplicate and included negative no-template controls. Primer pairs (**Table 1**) that spanned at least one intron-exon boundary and produced amplicons in the range of 100-200 bp, were designed using the Real-time PCR Primer Design software (VWR GenScript Corp., Piscatway, NJ), and synthesized (Sigma-Aldrich, ON). In addition, a primer pair for the housekeeping/reference gene GAPDH was also included. We verified the amplicon specificity and sensitivity of all primer pairs with PCR before applying to real-time PCR.

**Table 1:** Human primer sequences special designed for real-time RT-PCR amplification.

Gene	Forward/Reverse	Primer sequence	Accession #	Size of product (bp)	Melting temperature (°C)
PTHrP	F	5′ GGA GAC TGG TTC AGC AGT GG 3′	NM_002820	133	57.0
	R	5′ TTG TCA TGG AGG AGC TGA TG 3′	(all 4 variants)		54.0
GAPDH	F	5′ CAT GAG AAG TAT GAC AAC AGC CT 3′	NM_002046	113	55.0
	R	5′ AGT CCT TCC ACG ATA CCA AAG T 3′			56.0
Caspase-3	F	5′ CAT GGA AGC GAA TCA ATG GAC T 3′	NM_004346	139	56.0
	R	5′ CTG TAC CAG ACC GAG ATG TCA 3′			56.0
Caspase-9	F	5′ AGG ATT TGG TGA TGT CGG TG 3′	NM_001229	119	55.0
	R	5′ CAC GGC AGA AGT TCA CAT TG 3′			55.0

### Relative quantification using real-time PCR

GAPDH was designated as the reference gene for relative quantification, through which the expression of endogenous mRNA from GCT stromal cells was normalized. Cycle threshold (Ct) numbers were derived from the exponential phase of PCR amplification. Relative changes in mRNA expression were calculated using the comparative ΔΔCT (crossing point) method.

### Anti-PTHrP neutralization and PTHrP peptide rescue

Anti-PTHrP (1–34) neutralizing antiserum (T-4512, Bachem Americas, Inc., CA) was used to neutralize secreted PTHrP. Cells were seeded into 96 well plates at 1×103 cells/well in medium containing 10% FBS. After cell attachment (Day 0), cells were transferred to serum-free medium treated either with 10 µg/ml anti-PTHrP neutralizing antibody or 10 µg/ml IgG as a control. This concentration was determined in our preliminary study on the effect of toxicity and dosage response of the anti-PTHrP neutralizing antibody in stromal cells [10]. A range of 1 nM to 10 µM of PTHrP peptide (1–34, Bachem Americas, Inc., CA) was used to rescue cell death initiated by the neutralization of PTHrP. As a positive control, the classic apoptotic agent, etoposide (100 µM, Sigma Aldrich Canada Ltd., ON ) was used to demonstrate an apoptotic positive result. On Day 2, the cell culture medium was collected for the APO-1 apoptosis assay, and cells were studied in proliferation assays and flow cytometry for apoptosis and cell cycle analysis.

### WST-1 cell proliferation measurement

Mitochondrial dehydrogenase activity, as an indicator of cell number, was assessed by the mitochondrial-dependent reduction of WST-1 to formazan. At indicated time-points over a 2-day time course, 10 µl of the WST-1 premixed reagent (Roche Diagnostics, Quebec) were added to each well on a 96 well plate, and the plates were incubated at 37°C with 5% CO2 for 4 h, as recommended by the manufacturer. Each well was initially seeded with 1×104 cells. The plates were then placed on a shaker for 1 min, and the absorbance was determined in the CytoFluor Multi-Well Plate reader series 4000 (PerSeptive Biosystems) at 450 nm.

### Crystal violet cell proliferation measurement

The cytotoxic effect of anti-PTHrP was assessed by measuring the number of viable cells using crystal violet (CV). The CV cytotoxicity test is based on the inability of dead cells to remain adherent to cell culture plastic. As in the WST-1 cell proliferation assays, each well was initially seeded with 1×104 cells. Medium was removed from the 96 well plate and cells were washed two times with cold PBS and then incubated with 200 µl of 70% ethanol at 4°C for at least 30 min. After removing the ethanol, cells were incubated with 100 µl of a 0.1% CV solution for 30 min at room temperature. Excess CV was then removed by several washes with water. Cell culture plates were dried overnight. CV was dissolved in 33% acetic acid. The absorbance of the dissolved dye, corresponding to the number of viable cells, was measured in a microplate reader at 570 nm.

### APO-1/Fas Enzyme-linked immunosorbent (ELISA) assay

The concentration of soluble APO-1/Fas antigen in cell culture supernatants was measured in a quantitative ELISA (Invitrogen, ON). The ELISA was performed following the manufacturer's protocol. The concentration of soluble APO-1 was calculated using the absorbance reading at 450 nm, based on the results of a standard dilution curve.

### Caspase activity assay

Cells with various PTHrP treatments were seeded into a 96-well plate as described above. The Apo-ONE Homogeneous Caspase-3/7 assay (Promega Corp., WI) containing Z-DEVD-R110 was performed to quantitate caspase activity levels according to the manufacturer's protocol. Cells were incubated for four hours at ambient temperature prior to recording fluorescence (Ex  = 485 nm; Em  = 530 nm).

### Detection of apoptosis and cell cycle by flow cytometry

For apoptosis detection, cells were treated with anti-PTHrP neutralizing antiserum as described above and harvested at day 2. Cells were trypsinized and washed with PBS twice and then fixed with cold ethanol overnight. The extent of apoptosis was quantified by using the Annexin V-EGFP Apoptosis Detection Kit (GenScript USA Inc., NJ) as per the manufacturer's protocol. Most of the phosphatidylserines (PS) in the cell membrane phospholipids translocate to the outer surface during the early stages of apoptosis, and can be detected by staining with an enhanced green fluorescent protein (EGFP) fused with annexin V. In addition, propidium iodide (PI) is a nucleic dye, which can only pass through the membranes of cells in middle and late period of apoptosis and stain their nuclei red. Detection of annexin V-EGFP binding at early apoptosis was performed by flow cytometry (Ex  = 488 nm; Em  = 530 nm) using an FITC signal detector and PI staining at later stages of apoptosis by the phycoerythrin emission signal detector (Ex  = 488 nm; Em  = 620 nm). Flow cytometry was performed on the EPICS XL Cytometer (Beckman Coulter Canada, Inc., ON) equipped with 3-color fluorescence air-cooled 488 nm argon laser light source and detector range of 300 – 800 nm. The EXPO32 ADC software provides both acquisition and analysis for apoptosis.

For cell cycle analysis, the same treatment procedure, however with PI alone, was administered to cells. Flow cytometry was then performed using the PI detector and analyzed with a cell cycle specific protocol in the EXPO32 ADC software. A total of 10000 events were acquired and the cells were properly gated for both analyses.

### Detection of apoptosis by confocal microscopy

The cells were cultured on round glass coverslips in a 60 mm culture dish to about 70% confluency and subjected to anti-PTHrP neutralizing antiserum as described above. The coverslips were washed with PBS and fixed with cold ethanol overnight. The cells were then labeled with Annexin V-EGFP, PI and 4′,6-diamidino-2-phenylindole (DAPI) for 30 min in binding buffer at 37°C. The coverslips were washed with PBS and mounted. Stained nuclei and phophatidylserine were visualized and photographed using a Leica DM-IRB laser scanning confocal microscope (Leica Microsystem, Heidelberg, Germany). DAPI (excitation at 360 nm and emission at 456 nm), PI (excitation at 488 nm and emission at 620 nm) and EGFP (excitation at 488 nm and emission at 530 nm) were exited by the argon–krypton laser. Apoptosis was characterized by the morphological changes, namely, externalization of phosphatidylserine, cytoplasmic and nuclear shrinkage, chromatin condensation, and formation of apoptotic bodies.

### Statistical analysis

GraphPad Prism software (GraphPad Software, Inc., USA) was used for statistical analysis. All data are presented as mean ± standard error of the mean (SEM), and are representative of measurements that were performed on five different GCT patient samples (n = 5). To assess variations in real-time PCR gene expression, analysis of variance (ANOVA) and the post-hoc multiple comparison Tukey test (p<0.05) were applied. Measurements were normalized to the negative control. Each experiment was performed at least three times. P values <0.05 were considered to be statistically significant. The results of WST and crystal violet tests were presented as a percentage of the control value obtained in untreated cells.

## Results

### Expression of PTHrP in GCT stromal cells

**Figure 1:**
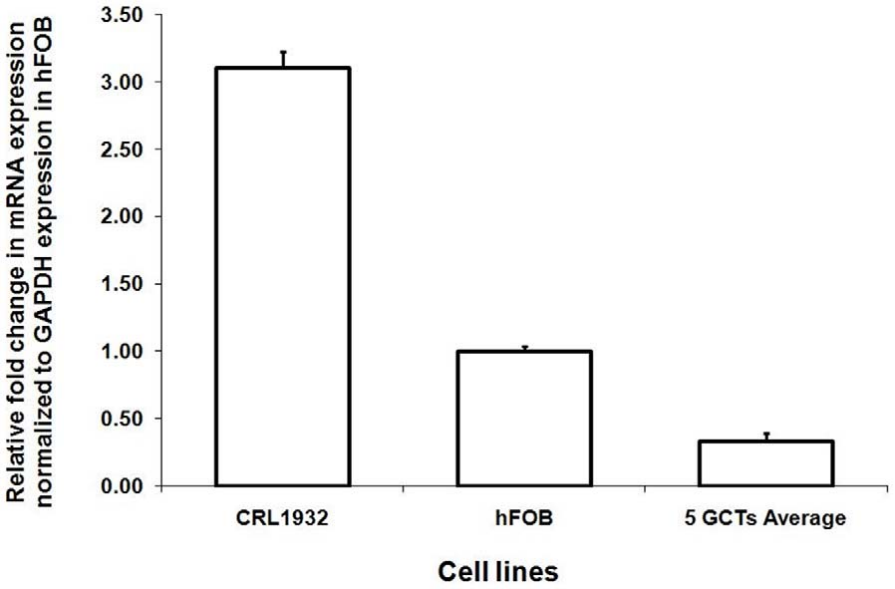
Relative mRNA expression of PTHrP based on real-time RT-PCR. The average expression of PTHrP in five different GCT stromal cells, CRL1932 and hFOB cells (controls). The ΔΔCT method was used to calculate the real-time RT-PCR fold change using GAPDH mRNA for normalization, and all changes in expression are relative to the control without any treatment. Triplicate independent real-time PCR experiments were performed.

To confirm the expression of PTHrP in GCT stromal cells, PTHrP mRNA was quantitated for CRL1932, hFOB and stromal cells of five GCT primary cell lines using real-time PCR. As expected, the positive control CRL1932 expressed a relatively high level of PTHrP (**Fig. 1**). Stromal cells of five GCT primary cell lines on average expressed a relatively low level of PTHrP mRNA. The expression of PTHrP protein by GCT stromal cells was previously reported using both immunohistochemistry and western blotting in our preliminary studies [10].

### The Effect of anti-PTHrP neutralization on GCT stromal cell morphology

**Figure 2:**
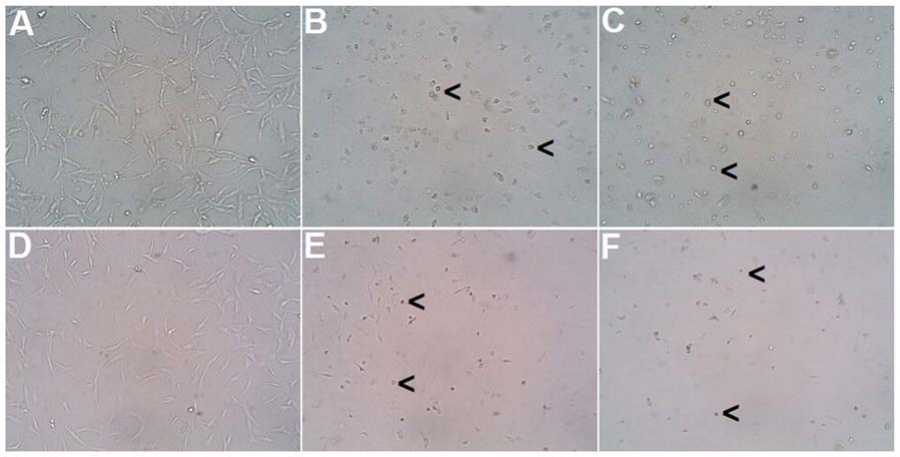
Cell morphology in the presence or absence of anti-PTHrP treatment. Cell morphology of renal cell adenocarcinoma CRL1932 (**A, B, C**) and GCT stromal cells (**D, E, F**) in the presence of IgG control (**A, D**), the apoptotic agent etoposide (**B, E**) and anti-PTHrP antiserum (**C, F**). Representative pictures were taken with light microscope at magnification ×200. Black arrows indicate examples of cells undergoing apoptosis.

To determine if blocking PTHrP activity would interfere with GCT cell growth, we administered 10 µg/ml of anti-PTHrP neutralizing antibody to cells. Both the CRL1932 cells and GCT stromal cells exposed to the PTHrP neutralizing antibody exhibited cytoplasmic shrinkage and formation of apoptotic bodies (**Figs. 2** **C and F**), similar to the apoptotic effects after treatment with the classic apoptotic agent, etoposide (**Figs. 2** **B and E**). Control cells exposed to IgG appeared healthy (**Figs. 2** **A and D**).

### Neutralizing PTHrP protein expression decreases cell proliferation

**Figure 3:**
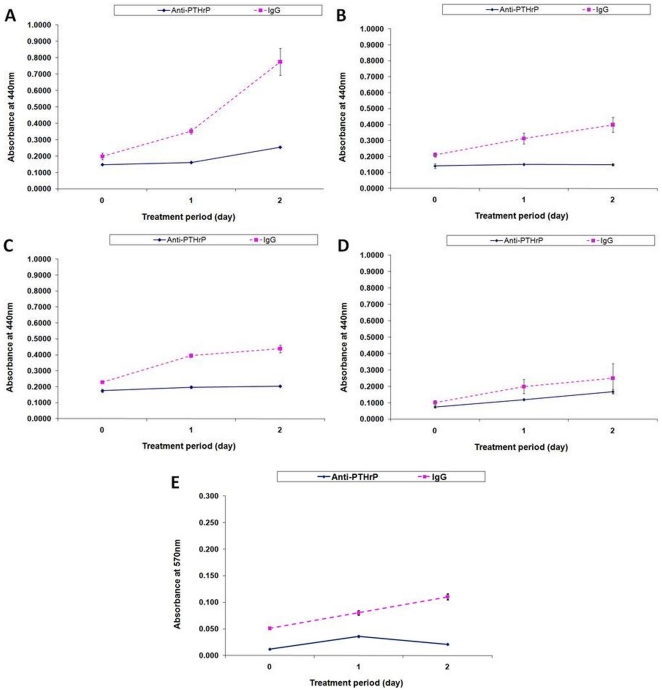
The effect of PTHrP neutralization on cell proliferation. Cells were treated with anti-PTHrP antibodies and assayed for cell growth using both WST-1 assay (**A**–**D**) and CV staining (**E**) over a two-day period. Total cell number was evaluated by measuring absorbance at 450 nm and 570 nm respectively. Cells assayed were **A**) CRL1932, **B**) hFOB, **C** & **E**) GCT stromal cells from primary bone and **D**) from a lung metastasis. Results are means ± SEM of triplicate independent experiments after normalized to the background control.

To measure the inhibitory effect of the anti-PTHrP neutralizing antibody on GCT cell proliferation, we used two separate approaches to assay cell viability. First, cell progression was assessed using the WST-1 assay. The cell growth of positive control CRL1932 cells was greatly reduced upon treatment with anti-PTHrP antibody on day 2 (**Fig. 3A**). Likewise, hFOB (**Fig. 3B**) and GCT stromal cells (**Fig. 3C**) decreased in cell proliferation on day 2 when treated with anti-PTHrP antibody. However, a subset of GCT stromal cells from a lung metastasis did not show any significant depletion in cell proliferation under anti-PTHrP treatment (**Fig. 3D**). Parallel experiments were conducted in a similar fashion and evaluated with CV staining. All cells demonstrated similar anti-PTHrP-induced growth reduction in the CV assay as compared to that of the WST-1 assay (only data from GCT stromal cells were shown in **Fig. 3E**). Cell progression in stromal cells from a GCT lung metastasis was also confirmed to be a subset within the CV assay in that cell proliferation was not affected by PTHrP neutralization (data not shown).

### The expression of of apoptotic markers

**Figure 4:**
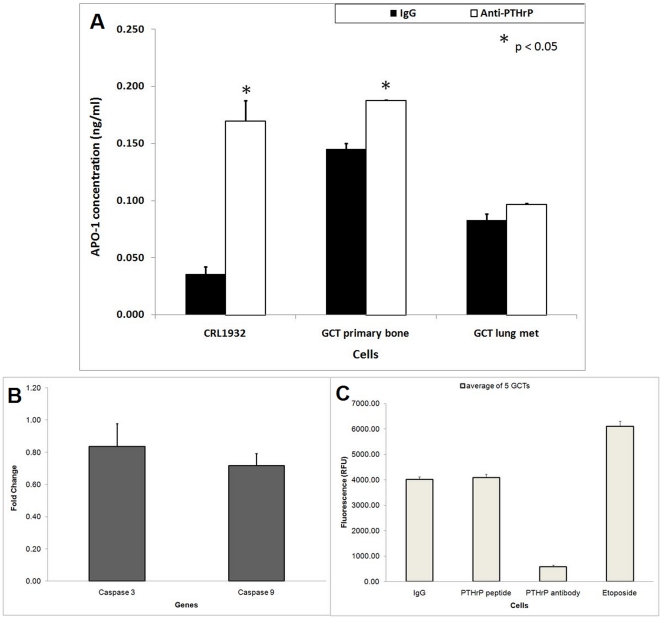
The effect of PTHrP on the expression of death markers. **A)** The effect of anti-PTHrP antibodies on the level of APO-1 concentration in CRL1932 and GCT bone and lung metastasis stromal cells. Filtered and concentrated conditioned media from cell cultures treated with and without anti-PTHrP antibodies were analyzed using the APO-1 ELISA kit as per optimization in our lab. Results of the ELISA assay in triplicate are shown by error bars. The APO-1 concentration levels were normalized to the amount of total protein for each condition. *P<.05 is versus corresponding IgG control values. Statistical comparison by analysis of variance with post hoc Tukey tests. **B)** Relative mRNA expression of caspase-3 and -9 based on real-time RT-PCR in five different GCT stromal cells. The ΔΔCT method was used to calculate the real-time RT-PCR fold change using GAPDH mRNA for normalization, and all changes in expression are relative to the control without any treatment. Triplicate independent real-time PCR experiments were performed. **C)** Caspase-3/7 activity assay was performed on GCT cells under various treatments in 96 well plate format. Average results of the activity assay of five GCT cells in triplicate are shown by error bars.

Soluble APO-1 (Fas/CD95) is a cell surface receptor that mediates apoptosis when it reacts with Fas ligand (FasL). We evaluated the effect of anti-PTHrP treatment on the levels of soluble APO-1 protein released in the culture medium using an APO-1 ELISA assay. As shown in **Fig. 4A**, CRL1932 cells secreted a higher APO-1 protein level in cell culture medium when treated with anti-PTHrP antibody. Similar increases in APO-1 levels were observed in the stromal cells from GCT. However, stromal cells from the GCT lung metastasis subgroup did not show any significant increases in APO-1 levels when exposed to PTHrP neutralization (**Fig. 4A**).

After the initiation of the cell death process mediated by the Fas/Apo-1 receptor, the activity of the caspase cascade is an important consideration. We examined both the genetic expression of caspases and caspase protein activity levels. To confirm the expression of caspase-3 and -9 mRNA in GCT stromal cells, both genes were quantitated for all five PTHrP-neutralized GCT primary cell lines using real time PCR. Unexpectedly, stromal cells treated with PTHrP antiserum on average expressed a lower level of caspase-3 and -9 mRNA (**Fig. 4B**). To further validate the involvement of caspase cascade at the protein activity level, the caspase-3/7 activity assay was performed. We confirmed that the classic apoptotic caspase pathway was not actively involved in the observed cell death when GCT cells were treated with PTHrP neutralization (**Fig. 4C**). Positive caspase activation was confirmed with etoposide, a DNA topoisomerase inhibitor.

### Detection of apoptosis using flow cytometry and confocal microscopy

**Figure 5:**
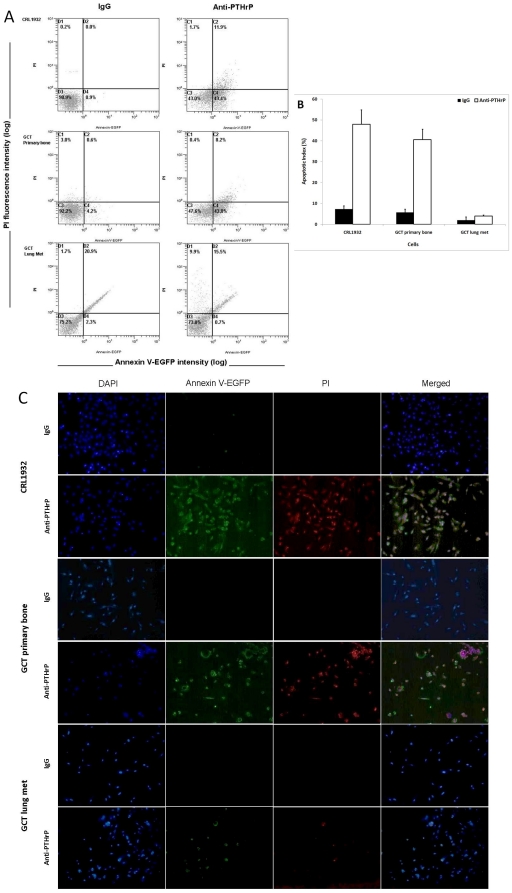
Apoptosis induced by the anti-PTHrP antibody immunotherapy. **A** & **B**) CRL1932 and GCT stromal cells and GCT lung metastasis were starvation synchronized and treated with either anti-PTHrP antiserum or IgG for two days. All cells were gated, and apoptosis was determined by Annexin V-FITC and PI staining with flow cytometry. Numbers within quadrants represent percentages of live (lower left quadrant), early apoptotic (lower right quadrant), and late apoptotic or necrotic (top left and right quadrant) cells. **A**) Representative results from FACS analysis showed apoptotic/necrotic distribution of proliferating CRL1932, GCT stromal cells from primary bone and lung metastasis with either anti-PTHrP antiserum or IgG. **B**) The average apoptotic index showing only the early apoptosis measurement for CRL1932 and GCT stromal cells from primary bone and a lung metastasis. Values represent the means ± SEM of triplicate independent experiments. **C**) Under the same treatment conditions, cells were grown on cover slips and underwent fluorescent staining with DAPI, annexin V-EGFP and PI for morphological determination of apoptosis and necrosis. Representative pictures were taken with corresponding filters using a fluorescent microscope.

The externalization of PS is an early event in apoptosis which could be determined using annexin V in flow cytometry. As a control, PI, a cell-impermeant nuclear stain, was used. The biparametric analysis of annexin V-EGFP versus PI of control cells with IgG was portrayed as a cytogram (**Fig. 5a** **, left column**). The majority of live cells were confined to the lower left quadrant of each cytogram indicating only a background level of PI and annexin V staining. Early apoptotic cells located in the lower right quadrant are annexin V positive and PI negative; late apoptotic or necrotic cells located in the upper right quadrant are PI positive and annexin V positive. Etoposide was used to show the classic apoptotic population distribution pattern on a cytogram (**Supplementary Figure S2**).

Treatment with anti-PTHrP antiserum increased the externalization of PS indicating early apoptosis in CRL1932 and GCT stromal cells as shown in **Fig. 5a** **, right column**. Stromal cells from a GCT lung metastasis showed a very slight gain in both annexin V and PI staining at the upper quadrants representing a small but insignificant increase in late apoptosis and necrotic cell death upon anti-PTHrP treatment. **Figure 5b** shows the average early apoptotic index with both CRL1932 and stromal cells from GCT undergoing early apoptosis in response to PTHrP neutralization. However, stromal cells from a GCT lung metastasis did not show a statistically significant increase in early apoptosis.

Identification of apoptotic cells by flow cytometry is generally based on a single feature by the molecular marker of apoptosis. Differential staining of cellular DNA and protein with DAPI, annexin V and PI of the cells on slides gives a very good morphological resolution of apoptosis and necrosis. The induction of apoptosis was confirmed by immunofluorescence microscopy, which showed chromatin condensation and apoptotic body formation. The results of microscopy confirmed the increase in early and late apoptotic cells in CRL1932 and GCT cells treated with anti-PTHrP antibodies with the exception of GCT cells from a lung metastasis (**Fig. 5c**).

### Cell cycle analysis by flow cytometry

**Figure 6:**
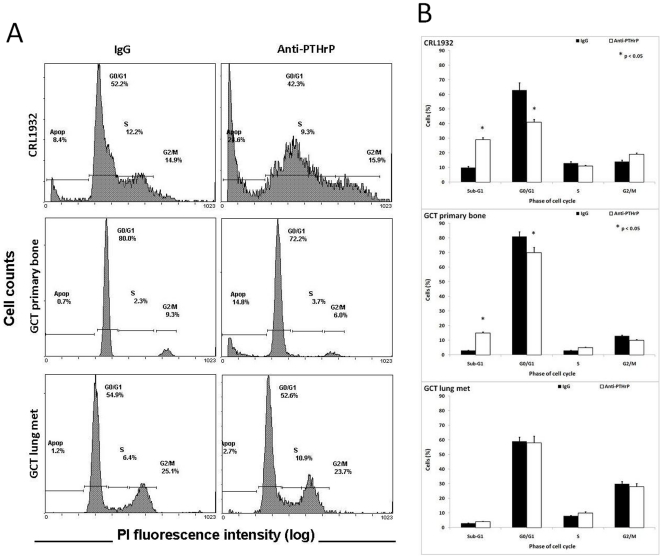
The effect of PTHrP on cell cycle phase distribution in proliferating cells. CRL1932, GCT stromal cells from primary bone and a lung metastasis were starvation synchronized and treated with either anti-PTHrP antiserum or IgG for two days. After fixing, cells were incubated with PI and analyzed with flow cytometry. (**A**) Representative results from FACS analysis showed cell cycle distribution of proliferating CRL1932, GCT stromal cells from primary bone and lung metastasis with either anti-PTHrP antiserum or IgG. (**B**) Percentage of cells in each cell cycle phase: Sub-G1, G0/G1, S and G2/M. Values represent the means ± SEM of triplicate experiments after normalized to the background control. *P<.05 is versus corresponding IgG control values. Statistical comparison by analysis of variance with post hoc Tukey tests.

To identify the cell cycle phase regulation of PTHrP, serum-starved cells were treated with or without anti-PTHrP antibody and harvested for flow cytometric analysis using the PI staining method. In contrast to the majority G0/G1 phase growth arrest in IgG treated control CRL1932 cells, anti-PTHrP treatment shifted the cell cycle progression to hypodiploid sub-G1 late apoptotic/necrotic phase (**Fig. 6A**). Insignificant changes were observed in the S and G2/M phases under anti-PTHrP treatment (**Fig. 6B**). Likewise, GCT stromal cells demonstrated a shift from G0/G1 to sub-G1 upon anti-PTHrP treatment in a lesser scale and also showed insignificant changes in the S and G2/M phases under anti-PTHrP treatment (**Figs. 6A, B**). Interestingly, no difference in cell cycle phase distribution to the sub-G1 phase was noted after anti-PTHrP treatment in stromal cells from a GCT lung metastasis (**Figs. 6A, B**). Again, treatment with etoposide showed a classic apoptotic population distribution pattern on the cell cycle histogram with a mild disturbance within each cell cycle phase (**Supplementary Figure S1**).

### PTHrP neutralization reversed by PTHrP peptide treatment

**Figure 7:**
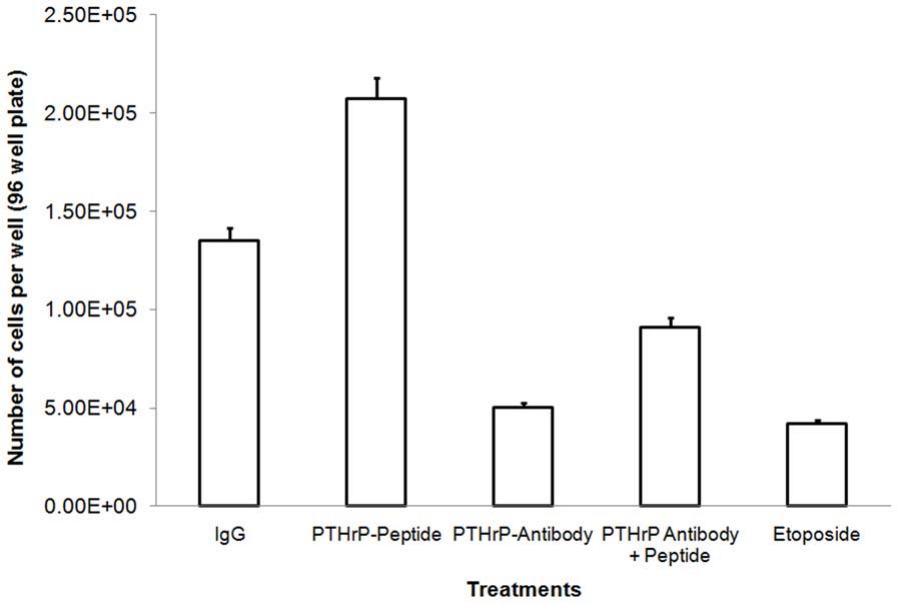
The reverse effect of PTHrP peptide treatment on cell proliferation. GCT stromal cells were treated with anti-PTHrP antibodies and PTHrP peptide. Each condition was assayed for cell growth using CV staining over a two-day period. Total cell number was evaluated by measuring absorbance at 570 nm. Results are means ± SEM of triplicate independent experiments after normalized to the background control.

To further identify whether PTHrP neutralization would solely contribute to the apoptotic phenomenon, we treated GCT stromal cells with both PTHrP antibody and PTHrP peptide to determine if ‘cell death rescue’ would occur. With incubation of both PTHrP antibody and PTHrP peptide, the number of proliferating GCT stromal cells increased significantly as compared to PTHrP antibody treatment alone (**Fig. 7** **& Supplementary Figure S3**). PTHrP peptide treatment increased proliferation as expected. Etoposide was a positive apoptotic indication.

## Discussion

PTHrP plays various important roles at multiple stages during bone formation through its effects on cell survival [22], [23], activation of growth factors, osteoblast cell proliferation [24], and mature osteoblast function [25]. Our recent findings that Runx2 and AP-1 is an important regulator in GCT stromal cells [7], [9] and that PTHrP is expressed by this tumor *in vivo* [10], together with the fact that Runx2 and PTHrP regulate each other in a reciprocal fashion through Ihh pathway [11], [12], [13], suggest that PTHrP may be one of the possible upstream or downstream effectors of Runx2. PTHrP has been shown to regulate tumor-relevant genes and to play a role in tumorigenesis, modulation of tumor progression and response to treatment in breast cancer and bone metastases [26], [27]. To elucidate the neoplastic role of PTHrP in GCT stromal cells, anti-PTHrP neutralizing antiserum was used to study the function of PTHrP in GCT cell proliferation and cell cycle progression.

In this study, we report that GCT stromal cells express a consistently modest level of PTHrP mRNA. The expression of PTHrP and PTHrP-receptors has also been confirmed with immunohistochemistry and western blotting in our preliminary data [10]. Our findings align with others regarding the presence of PTHrP in GCT cells [28], [29]. In the current study, both stromal cells of GCT and the classic PTHrP-secreting renal cell adenocarcinoma CRL1932 showed signs of apoptosis with cytoplasmic and nuclear shrinkage and formation of apoptotic bodies upon anti-PTHrP treatment. The renal cell adenocarcinoma data found in this study confirm the results obtained by Talon *et al*. in the same cell line [20].

The vital role of PTHrP in GCT cell growth was determined in the WST-1 and CV cell proliferation assays. Blockade of PTHrP ultimately halts the cell progression of stromal cells of GCT primary bone tumors. Unexpectedly, a subset of GCT stromal cells from a lung metastasis samples was identified based on its insignificant response toward the anti-PTHrP neutralizing antiserum. This intriguing result may be related to the specific cell microenvironment at the metastatic site and the distinct molecular signature required for survival in that environment [30]. However, this trend still needs to be validated with more lung metastatic samples.

The apoptosis induced by anti-PTHrP neutralizing antibody in GCT stromal cells was confirmed in this study with multiple approaches. Induction of apoptosis and activation of caspases can result from a variety of stimuli including growth factor withdrawal, exposure to radiation or chemotherapeutic agents, or initiation of the Fas/Apo-1 receptor-mediated cell death process. Increased concentration of the soluble APO-1 cell surface receptor that mediates apoptosis was observed after PTHrP neutralization. One of the direct mechanisms of apoptotic initiation in mammals is the Fas-Fas ligand-mediated model through APO-1. Apoptosis induced by PTHrP neutralization was equated by stimulation with etoposide, a DNA topoisomerase inhibitor that leads to caspase activation. Active caspases participate in a cascade of cleavage events that disable key homeostatic and repair enzymes and bring about systematic structural disassembly of dying cells. However, mitochondrial-mediated caspase dependent apoptosis was not observed in PTHrP antibody-treated GCT cells. PTHrP neutralization-induced cell death in GCT stromal cells may be a result of either direct signal transduction via Fas/APO-1 pathway or a caspase-independent apoptotic pathway that is mediated by apoptosis-inducing factor.

Moreover, flow cytometry for apoptosis showed a shift to the early apoptotic quadrant, as well as a shift to the subdiploid in cell cycle analysis under anti-PTHrP treatment. In addition, photos from light and immunofluorescent microscopy confirmed the apoptotic changes in physiology and morphology of GCT stromal cells when PTHrP was neutralized. All results led to the conclusion that PTHrP neutralization stimulates apoptosis in the neoplastic GCT stromal cells. Antagonistically, cell death induced by PTHrP neutralization is reversed by PTHrP peptide treatment, indicating the specificity of PTHrP action.

Apoptosis is the normal mechanism of cell turnover in embryonic development, metamorphosis and hormone-dependent atrophy of tissues [31]. Apoptosis exerts a homeostatic function in maintaining a balance between cell replication and cell death. Apoptotic cells are physiologically and morphologically different from necrotic cells. Characteristics of cell morphology when undergoing apoptosis included blebbing, loss of cell membrane asymmetry and attachment, cell shrinkage, nuclear fragmentation, chromatin condensation, and chromosomal DNA fragmentation [32]. Most tumor cells are prone to have a higher resistance against apoptosis and dysregulation of the cell as part of the neoplastic evolution [33]. Results from this study suggest the potential for anti-PTHrP treatment therapy in humans against GCT from primary bone sites.

Increased expression of PTHrP is associated with the development of bone metastasis in many tumors [19]. The question remains as to whether PTHrP would be a viable therapeutic target for primary bone tumors and bone metastases. Osteolysis caused by human breast cancer metastases was shown to be blocked by anti-PTHrP neutralizing antibodies [34]. Furthermore, a recent study has shown a therapeutic effect of anti-PTHrP treatment with zoledronic acid (a third-generation bisphosphonate) for bone metastases of small cell lung cancer in severe combined immunodeficient mice [35]. Similarly, immunization with anti-PTH antibodies was effective in controlling hypercalcemia for more than 72 months in patients with unresectable parathyroid carcinoma in Phase III clinical trials [36], [37].

Despite a significant number of studies that detailed the role of PTHrP in tumorigenesis, such studies generated as many questions as they answered [19]. Since the prognostic significance of PTHrP expression on primary tumor cells is an unsettled issue, any clinical utilities that manipulate PTHrP therapeutically need thoughtful consideration and understanding of the underlying mechanisms involved. Moreover, PTHrP might exert distinct effects on different cell microenvironments [38]. Antibodies against PTH/PTHrP, anti-RANKL therapy and bisphosphonates are at present some of the promising therapies that could be introduced into clinical trials; however, the underlying mechanisms of action should first be explored and clearly documented [35], [39], [40].

In conclusion, we demonstrated for the first time and with multiple assays that neutralization of PTHrP induces apoptosis in stromal cells of GCT. The direct corollary dictates that PTHrP serves to protect GCT stromal cells from apoptosis and therefore contributes a neoplastic phenotype. This data constitutes a starting point for clinical evaluation of anti-PTHrP strategies against primary GCT of bone. To further investigate this possibility, future studies exploring the mechanisms of apoptosis induced by anti-PTHrP immunotherapy will be vital. In addition, the concept that PTHrP serves to propagate proliferation in an autocrine manner in GCT stromal cells is an intriguing model for further investigation into this neoplastic phenomenon.

## Supporting Information

Figure S1 **The effect of PTHrP on cell cycle phase distribution in proliferating cells.** CRL1932 and GCT stromal cells were starvation synchronized and treated with either IgG vehicle, anti-PTHrP antiserum or the apoptotic agent etoposide for two days. After fixing, cells were incubated with PI and analyzed with flow cytometry. Representative results from FACS analysis showed cell cycle distribution of proliferating CRL1932 and GCT stromal cells with either IgG vehicle, anti-PTHrP antiserum or etoposide.
(TIF)

Figure S2 **Apoptosis induced by the anti-PTHrP antibody immunotherapy.** CRL1932 and GCT stromal cells were starvation synchronized and treated with either IgG vehicle, anti-PTHrP antiserum or the apoptotic agent etoposide for two days. All cells were gated, and apoptosis was determined by Annexin V-FITC and PI staining with flow cytometry. Numbers within quadrants represent percentages of live (lower left quadrant), early apoptotic (lower right quadrant), and late apoptotic or necrotic (top left and right quadrant) cells. Representative results from FACS analysis showed apoptotic/necrotic distribution of proliferating CRL1932 and GCT stromal cells with either IgG vehicle, anti-PTHrP antiserum or etoposide.
(TIF)

Figure S3 **Cell morphology in the presence of PTHrP peptide, antibody or both.** Cell morphology of GCT stromal cells in the presence of IgG control, PTHrP peptide, anti-PTHrP antiserum, and PTHrP peptide with antibody. Representative pictures were taken with light microscope at magnification ×200. Black arrows indicate examples of cells undergoing apoptosis.
(TIF)

## References

[1] Turcotte RE (2006). Giant cell tumor of bone.. Orthop Clin North Am.

[2] Balke M, Ahrens H, Streitbuerger A, Koehler G, Winkelmann W (2009). Treatment options for recurrent giant cell tumors of bone.. J Cancer Res Clin Oncol.

[3] Balke M, Schremper L, Gebert C, Ahrens H, Streitbuerger A (2008). Giant cell tumor of bone: treatment and outcome of 214 cases.. J Cancer Res Clin Oncol.

[4] Becker WT, Dohle J, Bernd L, Braun A, Cserhati M (2008). Local recurrence of giant cell tumor of bone after intralesional treatment with and without adjuvant therapy.. J Bone Joint Surg Am.

[5] Ghert M, Simunovic N, Cowan RW, Colterjohn N, Singh G (2007). Properties of the stromal cell in giant cell tumor of bone.. Clin Orthop Relat Res.

[6] Cowan RW, Mak IW, Colterjohn N, Singh G, Ghert M (2009). Collagenase expression and activity in the stromal cells from giant cell tumour of bone.. Bone.

[7] Mak IW, Cowan RW, Popovic S, Colterjohn N, Singh G (2009). Upregulation of MMP-13 via Runx2 in the stromal cell of Giant Cell Tumor of bone.. Bone.

[8] Mak IW, Seidlitz EP, Cowan RW, Turcotte RE, Popovic S (2010). Evidence for the role of matrix metalloproteinase-13 in bone resorption by giant cell tumor of bone.. Hum Pathol.

[9] Mak IW, Turcotte RE, Popovic S, Singh G, Ghert M (2011). AP-1 as a Regulator of MMP-13 in the Stromal Cell of Giant Cell Tumor of Bone..

[10] Cowan R, Singh G, Ghert M. The role of parathyroid hormone-related protein in giant cell tumour of bone.. Bone.

[11] Iwamoto M, Kitagaki J, Tamamura Y, Gentili C, Koyama E (2003). Runx2 expression and action in chondrocytes are regulated by retinoid signaling and parathyroid hormone-related peptide (PTHrP).. Osteoarthritis Cartilage.

[12] Ueta C, Iwamoto M, Kanatani N, Yoshida C, Liu Y (2001). Skeletal malformations caused by overexpression of Cbfa1 or its dominant negative form in chondrocytes.. J Cell Biol.

[13] Vortkamp A, Lee K, Lanske B, Segre GV, Kronenberg HM (1996). Regulation of rate of cartilage differentiation by Indian hedgehog and PTH-related protein.. Science.

[14] Burtis WJ (1992). Parathyroid hormone-related protein: structure, function, and measurement.. Clin Chem.

[15] Datta NS, Abou-Samra AB (2009). PTH and PTHrP signaling in osteoblasts.. Cell Signal.

[16] Philbrick WM, Wysolmerski JJ, Galbraith S, Holt E, Orloff JJ (1996). Defining the roles of parathyroid hormone-related protein in normal physiology.. Physiol Rev.

[17] Suva LJ, Winslow GA, Wettenhall RE, Hammonds RG, Moseley JM (1987). A parathyroid hormone-related protein implicated in malignant hypercalcemia: cloning and expression.. Science.

[18] Juppner H, Abou-Samra AB, Freeman M, Kong XF, Schipani E (1991). A G protein-linked receptor for parathyroid hormone and parathyroid hormone-related peptide.. Science.

[19] Nishihara M, Kanematsu T, Taguchi T, Razzaque MS (2007). PTHrP and tumorigenesis: is there a role in prognosis?. Ann N Y Acad Sci.

[20] Talon I, Lindner V, Sourbier C, Schordan E, Rothhut S (2006). Antitumor effect of parathyroid hormone-related protein neutralizing antibody in human renal cell carcinoma in vitro and in vivo.. Carcinogenesis.

[21] Thiede MA, Strewler GJ, Nissenson RA, Rosenblatt M, Rodan GA (1988). Human renal carcinoma expresses two messages encoding a parathyroid hormone-like peptide: evidence for the alternative splicing of a single-copy gene.. Proc Natl Acad Sci U S A.

[22] Manolagas SC (2000). Birth and death of bone cells: basic regulatory mechanisms and implications for the pathogenesis and treatment of osteoporosis.. Endocr Rev.

[23] Chen HL, Demiralp B, Schneider A, Koh AJ, Silve C (2002). Parathyroid hormone and parathyroid hormone-related protein exert both pro- and anti-apoptotic effects in mesenchymal cells.. J Biol Chem.

[24] Nishida S, Yamaguchi A, Tanizawa T, Endo N, Mashiba T (1994). Increased bone formation by intermittent parathyroid hormone administration is due to the stimulation of proliferation and differentiation of osteoprogenitor cells in bone marrow.. Bone.

[25] Qin L, Raggatt LJ, Partridge NC (2004). Parathyroid hormone: a double-edged sword for bone metabolism.. Trends Endocrinol Metab.

[26] Luparello C, Romanotto R, Tipa A, Sirchia R, Olmo N (2001). Midregion parathyroid hormone-related protein inhibits growth and invasion in vitro and tumorigenesis in vivo of human breast cancer cells.. J Bone Miner Res.

[27] Liao J, McCauley LK (2006). Skeletal metastasis: Established and emerging roles of parathyroid hormone related protein (PTHrP).. Cancer Metastasis Rev.

[28] Nakashima M, Nakayama T, Ohtsuru A, Fukada E, Niino D (2003). Expression of parathyroid hormone (PTH)-related peptide (PthrP) and PTH/PTHrP receptor in osteoclast-like giant cells.. Pathol Res Pract.

[29] Kartsogiannis V, Udagawa N, Ng KW, Martin TJ, Moseley JM (1998). Localization of parathyroid hormone-related protein in osteoclasts by in situ hybridization and immunohistochemistry.. Bone.

[30] Wikman H, Vessella R, Pantel K (2008). Cancer micrometastasis and tumour dormancy.. APMIS.

[31] Blank M, Shiloh Y (2007). Programs for cell death: apoptosis is only one way to go.. Cell Cycle.

[32] Kanduc D, Mittelman A, Serpico R, Sinigaglia E, Sinha AA (2002). Cell death: apoptosis versus necrosis (review).. Int J Oncol.

[33] Klein G (2004). Cancer, apoptosis, and nonimmune surveillance.. Cell Death Differ.

[34] Guise TA, Kozlow WM, Heras-Herzig A, Padalecki SS, Yin JJ (2005). Molecular mechanisms of breast cancer metastases to bone.. Clin Breast Cancer 5 Suppl.

[35] Yamada T, Muguruma H, Yano S, Ikuta K, Ogino H (2009). Intensification therapy with anti-parathyroid hormone-related protein antibody plus zoledronic acid for bone metastases of small cell lung cancer cells in severe combined immunodeficient mice.. Mol Cancer Ther.

[36] Bradwell AR, Harvey TC (1999). Control of hypercalcaemia of parathyroid carcinoma by immunisation.. Lancet.

[37] Betea D, Bradwell AR, Harvey TC, Mead GP, Schmidt-Gayk H (2004). Hormonal and biochemical normalization and tumor shrinkage induced by anti-parathyroid hormone immunotherapy in a patient with metastatic parathyroid carcinoma.. J Clin Endocrinol Metab.

[38] Henderson MA, Danks JA, Slavin JL, Byrnes GB, Choong PF (2006). Parathyroid hormone-related protein localization in breast cancers predict improved prognosis.. Cancer Res.

[39] Thomas DM, Skubitz KM (2009). Giant cell tumour of bone.. Curr Opin Oncol.

[40] Tse LF, Wong KC, Kumta SM, Huang L, Chow TC (2008). Bisphosphonates reduce local recurrence in extremity giant cell tumor of bone: a case-control study.. Bone.

