# Supplementary figures and images for: PTHrP Induces Autocrine/Paracrine Proliferation of Bone Tumor Cells through Inhibition of Apoptosis
Source: PLoS One. 2011 May 23;6(5):e19975. doi: 10.1371/journal.pone.0019975 (PMC3100318; doi:10.1371/journal.pone.0019975)

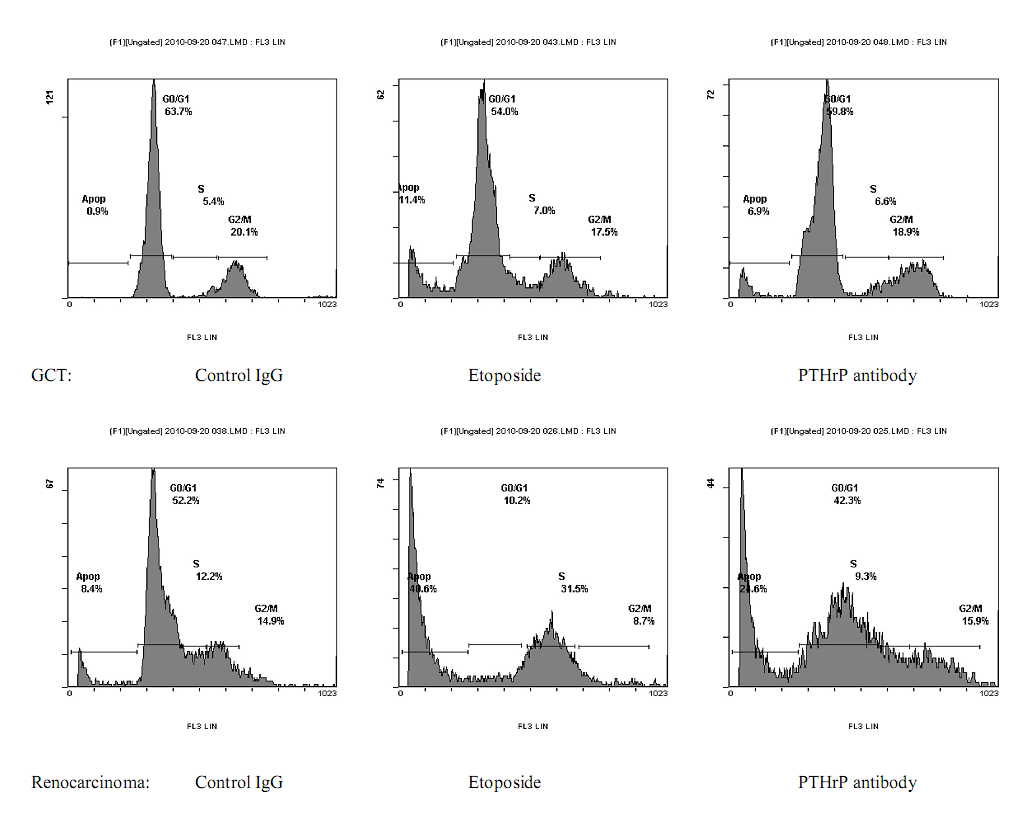

Supplement: Figure S1 — The effect of PTHrP on cell cycle phase distribution in proliferating cells. CRL1932 and GCT stromal cells were starvation synchronized and treated with either IgG vehicle, anti-PTHrP antiserum or the apoptotic agent etoposide for two days. After fixing, cells were incubated with PI and analyzed with flow cytometry. Representative results from FACS analysis showed cell cycle distribution of proliferating CRL1932 and GCT stromal cells with either IgG vehicle, anti-PTHrP antiserum or etoposide. (TIF) [file pone.0019975.s001.tif]

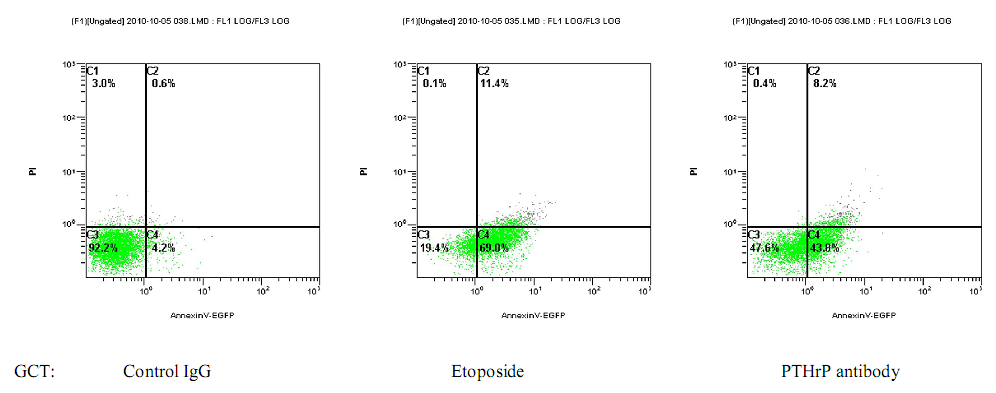

Supplement: Figure S2 — Apoptosis induced by the anti-PTHrP antibody immunotherapy. CRL1932 and GCT stromal cells were starvation synchronized and treated with either IgG vehicle, anti-PTHrP antiserum or the apoptotic agent etoposide for two days. All cells were gated, and apoptosis was determined by Annexin V-FITC and PI staining with flow cytometry. Numbers within quadrants represent percentages of live (lower left quadrant), early apoptotic (lower right quadrant), and late apoptotic or necrotic (top left and right quadrant) cells. Representative results from FACS analysis showed apoptotic/necrotic distribution of proliferating CRL1932 and GCT stromal cells with either IgG vehicle, anti-PTHrP antiserum or etoposide. (TIF) [file pone.0019975.s002.tif]

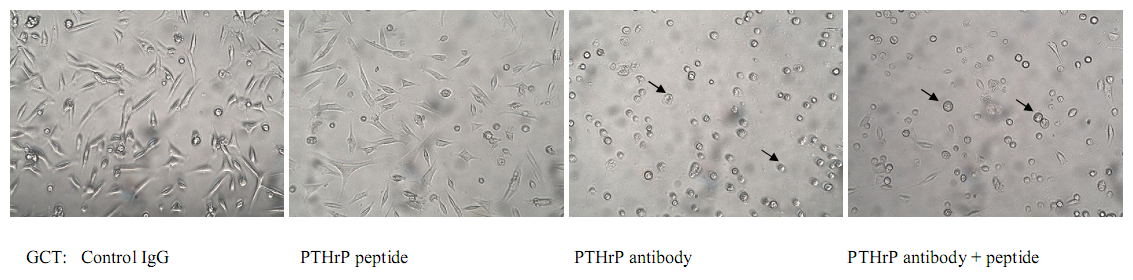

Supplement: Figure S3 — Cell morphology in the presence of PTHrP peptide, antibody or both. Cell morphology of GCT stromal cells in the presence of IgG control, PTHrP peptide, anti-PTHrP antiserum, and PTHrP peptide with antibody. Representative pictures were taken with light microscope at magnification ×200. Black arrows indicate examples of cells undergoing apoptosis. (TIF) [file pone.0019975.s003.tif]
